# A novel treatment of hyperinsulinemic hypoglycemia induced by insulin antibodies with alkali administration: a case report

**DOI:** 10.1186/s13256-019-1989-8

**Published:** 2019-03-11

**Authors:** Seiji Kobayashi, Houichi Amano, Yoshindo Kawaguchi, Takashi Yokoo

**Affiliations:** 1 Department of Allergy and Rheumatology, Nippon Medical School Graduate School of Medicine, 1-1-5 Sendagi, Bunkyo-ku, Tokyo, 113-8603 Japan; 2 Division of Nephrology and Hypertension, Department of Internal Medicine, The Jikei University School of Medicine, Tokyo, Japan; 3 Graduate School of Public Health, Teikyo University, Tokyo, Japan

**Keywords:** Insulin autoimmune syndrome, Insulin antibodies, Sodium bicarbonate, Acidosis

## Abstract

**Background:**

Insulin autoimmune syndrome is a rare cause of hyperinsulinemic hypoglycemia characterized by autoantibodies to human insulin without previous insulin use. We report a case of a patient with hyperinsulinemic hypoglycemia possibly caused by insulin antibodies induced by insulin analogs and a novel therapeutic measure for this condition.

**Case presentation:**

An 84-year-old Japanese man with a 28-year history of type 2 diabetes and chronic kidney disease, treated with biphasic insulin aspart 30, experienced persistent early morning hypoglycemia with daytime hyperglycemia. Despite discontinuation of biphasic insulin aspart 30, the condition persisted even after the patient ate small, frequent meals. Sodium bicarbonate was administered to correct the chronic metabolic acidosis, which then rectified the early morning glucose level.

**Conclusions:**

We believe this to be the first published case of a therapeutic approach to the treatment of hyperinsulinemic hypoglycemia associated with insulin antibodies that factors in blood pH and the correction of acidosis using sodium bicarbonate, which physicians could consider.

## Background

Insulin autoimmune syndrome (IAS) is a rare cause of hyperinsulinemic hypoglycemia characterized by high titers of autoantibodies to human insulin in individuals without previous insulin use [1]. The antibodies in IAS have lower affinity and higher capacity than non-IAS antibodies. Although several therapeutic approaches to IAS have been reported, the optimal treatment is yet to be found [2]. We report a case of a patient with hyperinsulinemic hypoglycemia possibly caused by insulin antibodies induced by insulin analogs and a novel therapeutic measure for this condition.

## Case presentation

An 84-year-old Japanese man was diagnosed with type 2 diabetes at 58 years of age in 1987. He received human insulin treatment for 20 years, but in 2011, biphasic human insulin 30 was changed to biphasic insulin aspart 30 (BIAsp 30). He had stage 4 chronic kidney disease due to nephrosclerosis, renal anemia, hypertension, dyslipidemia, hyperuricemia, and sleep apnea syndrome. He had been taking the following medications: amlodipine 10 mg/day, rosuvastatin 2.5 mg/day, and febuxostat 20 mg/day. He drank alcohol occasionally and had smoked one to two packs of cigarettes daily for 50 years when he quit 15 years ago. He did not have any food or drug allergies. His family and social histories were not remarkable. His environmental history revealed no abnormalities. He was a retired company director. From January 2015, he experienced persistent early morning hypoglycemia (< 50 mg/dl) with daytime hyperglycemia. Despite reduction of BIAsp 30 dosage, early morning hypoglycemia concomitant with disturbance of consciousness continued to occur. Therefore, he was admitted to our hospital in February 2015.

On examination, the patient’s temperature was 36.3 °C, pulse 64 beats/min, blood pressure 126/72 mmHg, respiratory rate 20 breaths/min, and oxygen saturation 96% while breathing ambient air. He was alert and oriented to time and place on admission. Neurological examination revealed intact cranial nerves, normal limb power and sensation, and absence of cerebellar signs. No changes in sensorium or psychotic features were noted. Other physical examinations revealed no abnormalities. Laboratory findings on admission were as follows: fasting plasma glucose, 82 mg/dl; hemoglobin A1c (HbA1c), 7.0%; and glycoalbumin, 21.4%. More laboratory test results are shown in Table 1. Imaging studies, including computed tomography and magnetic resonance imaging, showed no significant change.

**Table 1:** Laboratory data on admission

Laboratory test	Reference range	On admission
Blood		
Red blood cells (/μl)	430–540 × 10^4^	313 × 10^4^
Hemoglobin (g/dl)	14.0–18.0	10.7
White blood cells (/μl)	4000–8000	4500
Platelet count (/μl)	15–35 × 10^4^	18.6 × 10^4^
Reticulocytes (%)	0.7–2.0	0.8
Plasma glucose level (mg/dl)	70–110	82
HbA1c (%)	4.9–6.0	7.0
Glycoalbumin (%)	12.4–16.3	21.4
Aspartate aminotransferase (U/L)	8–38	13
Alanine aminotransferase (U/L)	4–44	9
Lactate dehydrogenase (U/L)	106–211	213
Alkaline phosphatase (U/L)	104–338	142
γ-Guanosine triphosphate (U/L)	16–73	31
Amylase (U/L)	40–129	81
Total protein (g/dl)	6.7–8.3	7.2
Albumin (g/dl)	3.8–4.9	4.1
Urea nitrogen (mg/dl)	8–20	33.4
Creatinine (mg/dl)	0.6–1.2	2.17
Uric acid	4–7	7.1
Sodium (mEq/L)	135–148	145
Potassium (mEq/L)	3.6–5.0	5.3
Chloride (mEq/L)	98–108	114
Fe (μg/dl)	65–157	80
Total iron-binding capacity (μg/dl)	256–426	289
Ferritin (ng/ml)	17–291	45.6
Total cholesterol (mg/dl)	120–220	103
Low-density lipoprotein cholesterol (mg/dl)	40–119	63
High-density lipoprotein cholesterol (mg/dl)	42–67	40.1
Triglycerides (mg/dl)	30–150	40
C-reactive protein (mg/dl)	0.3	0.05
Immunoreactive insulin (μIU/ml)	2.2–12.4	> 600
Free triiodothyronine (pg/ml)	1.71–3.71	2.70
Free thyroxine (ng/dl)	0.70–1.48	1.10
Thyroid-stimulating hormone (μIU/ml)	0.35–4.94	2.05
Cortisol (μg/dl)	6.2–19.4	17.4
Adrenocorticotropic hormone (pg/ml)	7.2–63.3	23.8
Adrenalin (pg/ml)	< 100	79
Noradrenalin (pg/ml)	100–450	913
Dopamine (pg/ml)	< 20	22
Glucagon (pg/ml)	70–174	182
Insulin-like growth factor 1 (ng/ml)	44–177	63
Growth hormone (ng/ml)	< 2.47	0.61
Insulin antibodies (U/ml)	< 0.4	> 50.0
Anti-glutamic acid decarboxylase antibody (U/ml)	< 1.5	0.9
Anti-islet cell antibody (U)	< 1.25	< 1.25
Anti-insulinoma-associated 2 antibody (U/ml)	< 0.4	< 0.4
Urine		
pH	5.0–9.0	5.5
Specific gravity	1.001–1.035	1.022
Protein	Negative	Negative
Glucose	Negative	Negative
Ketone	Negative	Negative
Albumin-to-creatinine ratio (mg/g Cr)	< 150	34.8
C-peptide immunoreactivity (μg/day)	29.2–167	40.5
Arterial blood gas		
pH	7.35 ± 7.45	7.343
Partial pressure of carbon dioxide (mmHg)	32–46	26.7
Partial pressure of oxygen (mmHg)	74–108	92.5
Carbon dioxide (mmol/L)	21–29	14.2
Base excess (mmol/L)	− 4	− 10.1
Oxygen saturation	92–96	96.4
Human leukocyte antigen DNA typing		DRB1*04:05, DRB1*08:03:02

*Cr* Creatinine, *HbA1c* Hemoglobin A1c

Although BIAsp 30 was discontinued after admission, early morning hypoglycemia with daytime hyperglycemia continued even after eating small frequent meals (a four or six meals per day eating pattern). Fasting blood samples revealed a plasma glucose level of 28 mg/dl, immunoreactive insulin > 2000 μIU/ml, C-peptide 3.03 ng/ml, and high titers of insulin antibody (IA) (> 50 U/ml). IA binding rate was at a high level (86.3%). Scatchard analysis showed an affinity contact (K1) of 0.00256 × 10^8^ M^− 1^ and a binding capacity (B1) of 99.7 × 10^− 8^ M against human insulin for the high-affinity sites, indicating that the patient’s IA bound to insulin with low affinity and high binding capacity. He had no history of medication involving SH residues or supplements containing α-lipoic acid. Moreover, workup for endocrinological abnormality and autoimmune disease did not reveal any significant findings (Table 1). HLA-DRB1*04:06 was undetectable, and imaging studies of the head and abdomen showed no evidence of abnormalities.

**Fig. 1:**
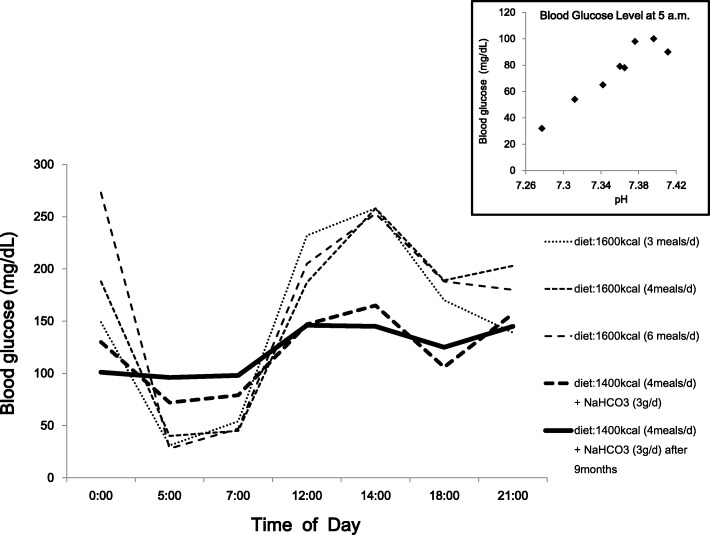
Blood glucose levels in each eating pattern with or without alkali administration. Changes in plasma glucose levels were monitored at indicated times (0:00, 5:00, 7:00, 12:00, 14:00, 18:00, 21:00) in each eating pattern with or without administration of sodium bicarbonate. The inset shows plasma glucose level at 5:00 a.m. after raising the arterial pH to 7.4 by administration of sodium bicarbonate

The patient’s serum creatinine level was 2.17 mg/dl, and his estimated glomerular filtration rate (eGFR) was 23.3 ml/min/1.73 m^2^. His arterial pH at 5:00 a.m. was 7.277, bicarbonate was 15.1 mEq/L, and base excess was − 10.7. After he was given a gradually increasing dose up to 3 g/day of sodium bicarbonate (split four times per day) for the purpose of correcting metabolic acidosis, his early morning glucose level was improved, concurrently bringing pH up to 7.4 (Fig. 1). Early morning hypoglycemia disappeared after he took 3 g/day of sodium bicarbonate and three meals plus snacks at night daily (1400 kcal/day) without any oral hypoglycemic agent or insulin. The patient was discharged in late March 2015 and continued on the same treatment.

After 9 months of follow-up with these treatments, the patient’s plasma glucose level at 5:00 a.m. was 96 mg/dl, and his arterial pH was 7.376. His immunoreactive insulin level had significantly decreased to 11.4 μIU/ml, even though the titer of IA remained high (> 50 U/ml). IA binding rate decreased to 42.1%. According to the Scatchard analysis, his IA shifted to higher affinity (K1 = 0.142 × 10^8^ M^− 1^) and lower capacity (B1 = 0.969 × 10^− 8^ M) than his previous IA. During this follow-up period, he had no symptoms of hypoglycemia, his HbA1c levels were around 6.5%, and his eGFR did not change significantly. His daily plasma glucose levels ranged from 96 to 168 mg/dl.

## Discussion and conclusions

We report a case of a patient with hyperinsulinemic hypoglycemia possibly caused by IA induced by insulin analogs that had lower affinity and higher capacity against insulin. IA are often detected in patients undergoing insulin treatment and rarely cause hyperglycemia or hypoglycemia, because these antibodies usually have low capacity or high affinity. However, IA in IAS have lower affinity and higher capacity against insulin for the high-affinity sites than non-IAS antibodies [3]. Our patient’s case was analogous to IAS, whereas he produced IA that had lower affinity and higher capacity than those reported in typical IAS cases.

The widely accepted hypothesis for pathophysiology in IAS is as follows: massive volumes of insulin binding to IA causing postprandial hyperglycemia to persist and the release of insulin from immunocomplexes triggering hypoglycemia. However, the mechanism by which insulin binding occurs during the day and dissociation occurs in the early morning is unknown. The study of the effect of different pH values on insulin-binding capacity of IA showed that IA from patients with high titers of IA (> 40%) dissociated from insulin in lower pH, whereas this phenomenon was not observed in patients with low titers of IA (< 20%) [4]. In our patient, sodium bicarbonate was administered to correct the chronic metabolic acidosis, which then rectified the early morning glucose level. We propose that one possible mechanism for hypoglycemia in IAS is dissociation of IA from insulin in individuals with metabolic and/or respiratory acidosis in the early morning. However, many details of the overarching mechanism remain to be elucidated.

Small, frequent meals remain the first line of treatment for IAS, and patients with severe hypoglycemia require adjunct therapy, such as glucocorticoid therapy, which suppresses the production of antibodies and plasmapheresis, which reduce IA titers [5]. Recently, it was reported that hypoglycemia due to IAS was successfully managed with rituximab [6]. However, immunosuppressive therapies may be accompanied by adverse events, particularly infections, especially in the elderly. Our patient was successfully treated with sodium bicarbonate and frequent meals in small quantities.

We believe this to be the first published case report of a therapeutic approach to hyperinsulinemic hypoglycemia resembling IAS, where blood pH seems to have had a pivotal role. Physicians should consider correction of acidosis using sodium bicarbonate as one option for the treatment of hyperinsulinemic hypoglycemia associated with IA.

## Abbreviations

BIAsp 30: Biphasic insulin aspart 30
eGFR: Estimated glomerular filtration rate
HbA1c: Hemoglobin A1c
IA: Insulin antibody
IAS: Insulin autoimmune syndrome
